# Para-Aortic Lymph Node Dissection and Metastasis Increase the Rate of Postoperative VTE in Gynaecological Cancers

**DOI:** 10.3390/cancers18010040

**Published:** 2025-12-22

**Authors:** Elzahra Ibrahim, Sharoon O’Toole, Lucy Norris, Feras Abu Saadeh

**Affiliations:** 1 Division of Gynaecology Oncology, St. James’s Hospital, D08 NHY1 Dublin, Ireland; fabusaad@tcd.ie; 2 Department of Obstetrics and Gynaecology, Trinity Centre for Health Sciences, St. James’s Hospital, D08 NHY1 Dublin, Ireland; shotoole@tcd.ie (S.O.); lnorris@tcd.ie (L.N.); 3 Trinity St. James’s Cancer Institute, St. James’s Hospital, D08 NHY1 Dublin, Ireland

**Keywords:** lymphadenectomy, pelvic lymphadenectomy, para-aortic lymphadenectomy, thrombosis, thromboprophylaxis, gynaecology cancer

## Abstract

**Objective:** We investigated the relationship between venous thromboembolism (VTE) and pelvic and para-aortic lymphadenectomy (LND) within the first 90 days post gynaecological cancer surgery. **Methods:** A retrospective cohort analysis was conducted on 1021 patients who underwent gynaecological cancer surgery between 2006 and 2019. Univariate and multivariate analysis was performed to assess the effects of LND and lymph node (LN) metastasis on VTE occurrence within 90 days post-surgery. **Results:** A total of 41 patients developed VTE within 90 days post-surgery. Para-aortic LND was significantly associated with VTE (*p* < 0.001), with the highest rates (14.6%) observed in patients who had >10 para-aortic LN removed. In patients with pelvic LN metastasis, 10 (7.5%) developed VTE; patients with >5 pelvic nodes positive for metastasis had an HR = 4.83 (95% CI: 0.99–13.9) after adjustment for age, duration of hospital stay, and surgical approach. The highest VTE rates (27.3%) occurred in patients with >5 para-aortic LN positive for metastasis, HR = 3.79 (95% CI 1.44–14.23) after adjustment for age, duration of hospital stay, and surgical approach (*p* = 0.011). **Conclusions:** Para-aortic LND significantly increases VTE risk within the first 90 days post-surgery. The risk is further amplified in cases with metastatic LN and persists even after adjustment for other risk factors for VTE. While extended thromboprophylaxis is standard for all cancer patients, our findings suggest that para-aortic LND—especially with nodal metastases—may help identify those who would benefit most from a more tailored, risk-based prophylaxis approach.

## Simple Summary

Cancer is associated with an increased risk of thrombosis; this is one of the main causes of mortality after cancer. Surgery is associated with increased VTE; this risk increases with the complexity of surgery. LND is part of gynaecology cancer surgery, involving mainly pelvic or para-aortic areas which add to the surgical complexity. We studied the effect of pelvic and para-aortic LND on the risk of VTE. We concluded that VTE is higher in gynaecological cancer patients following para-aortic LND, especially when more than 10 nodes were removed. Pelvic LND was not associated with an increased risk of VTE post cancer surgery. Para-aortic and pelvic LN metastasis status significantly increases the risk of postoperative VTE in gynaecological cancers. LN metastasis may be a potential predictive marker for postoperative VTE. These findings highlight the importance of thromboprophylaxis in gynaecology cancer patients’ post-surgery, especially in patients undergoing para-aortic LND.

## 1. Introduction

Venous thromboembolism (VTE) is a major cause of morbidity and mortality in gynaecological cancer patients and is the leading cause of death after the cancer itself [1]. The risk of VTE in gynaecological cancer differs according to the site and type of cancer, ranging from 6% in endometrial cancers to 43% in clear cell cancer of the ovary [2,3,4]. Cancer surgery is a major contributor to VTE risk; the first postoperative week is the peak time for VTE, with 75% of VTE occurring in the first 7 days due to the direct effect of surgery [5], while VTE occurring from day 8 till day 90 is attributed to the effect of recovery from surgery and adjuvant treatment. Gynaecological cancer surgery can be complex and may involve an extended hospital stay, which contribute to the risk of postoperative VTE [5]. For this reason, extended thromboprophylaxis for 28 days with low molecular weight heparin (LMWH) is recommended for all patients undergoing major pelvic-abdominal surgery for cancer [6]. Meta-analysis has shown that this approach is successful in significantly reducing VTE risk post-surgery [6,7]. Observational studies, particularly in endometrial cancer, suggest that the risk of surgery following minimally invasive surgery is low (0.57%) and does not support the use of extended thromboprophylaxis. This suggests that risk assessment is required to identify cases which may benefit from extended prophylaxis [8,9,10].

Lymph node dissection (LND) is an important part of gynaecological cancer surgery and involves the pelvic with or without para-aortic lymph nodes (LNs) [11]. LND is commonly performed in endometrial, vulvar, and cervical cancer surgeries and is important in planning adjuvant treatment, particularly in endometrial cancer. The role of routine LND in ovarian cancer is controversial; systemic LND does not improve overall survival [12,13]. According to the LION (lymphadenectomy in ovarian neoplasms) study, systematic LND in advanced ovarian cancer with clinically negative lymphadenopathy is associated with higher morbidity and mortality [14]. Sentinel LND is becoming a more common practice to reduce risks of LND, especially in endometrial, cervical, and vulval cancers [11,15]. In the early stages of cervical cancer, SLN and frozen section is the recommended approach; in the case of positive LN, surgery is abandoned [16]. SLND is to be performed for all cases of endometrial cancer with no metastasis or LN involvement [17].

In a large study of mixed cancer types, LN metastasis was reported to have a strong association with increased VTE risk [18]. The risk varies depending on the type of cancer [19,20]. In a large study of prostate cancer, patients who had LND had a significantly higher risk of VTE (6–8-fold) compared with those who did not require the procedure. [21]. In a large study of early-stage endometrial cancer (stage I–II) [20], LND increased the postoperative VTE rate. Patients who underwent open surgery had a VTE rate of 4.3% compared with those who had minimally invasive surgery (MIS), who had a VTE rate of 2.87%. However, the effect of different LND sites on the risk of VTE in gynaecological cancer patients was not explored.

In this study, we investigated the relationship between postoperative VTE risk and the site of LND (pelvic and para-aortic) in gynaecological cancer patients. We also examined the impact of lymph node metastasis on the risk of VTE within the first 90 days following gynaecological cancer surgery.

## 2. Materials and Methods

This study was a retrospective cohort study that used data collected as part of the Trinity College Dublin (TCD) Gynaecological Cancer Bioresource in Dublin, Ireland. Patients who underwent gynaecological cancer surgery (excluding diagnostic procedures) in St. James Hospital, Dublin, between January 2006 and June 2019 and who consented to take part in the Trinity College Gynaecological Cancer Bioresource were included in the study. The TCD gynaecological cancer bioresource is an extensive bioresource of serum, blood, and plasma from gynaecological cancer patients which has been running since 2004. The bioresource invites patients undergoing treatment for gynaecological cancer in St. James’s Hospital (SJH) gynaecology–oncology unit (a tertiary referral centre) in Dublin, Ireland, to donate blood, tissue, and medical data for research. All patients participating in the biobank gave full and informed written consent, and the biobank and the resulting studies are approved by the Tallaght University Hospital/St. James’s Hospital Joint Research Ethics Committee. Blood samples are obtained preoperatively. All clinicopathological details are extracted from the hospital records, and patient follow-up is recorded on a dedicated database. Diagnostic histology and radiology are reviewed by the multidisciplinary group at the tumour board at our tertiary cancer care centre prior to and after surgical staging. Patients with prior documented VTE within the last 5 years were excluded from the study. Also, patients with any history of significant haemorrhage outside of a surgical setting within the last 5 years, familial bleeding diathesis, or currently receiving long-term anticoagulant therapy were excluded.

Since June 2012, all gynaecological cancer patients undergoing cancer surgery (including both open and MIS approaches) routinely have received postoperative thromboprophylaxis for 28 days post-surgery in accordance with the guidelines [22]. In 2012, the change in practice was implemented after the emerging recommendation from the American College of Obstetric and Gynaecology (ACOG) and systemic review articles [23,24]. Patients with a BMI < 40 were prescribed 4500 IU of low molecular weight heparin (LMWH) (Tinzaparin) once daily, while those with a BMI ≥ 40 were prescribed a weight-adjusted dose of 75 IU/kg daily. Anti-embolic compression stocking was provided to all postoperative patients during their hospital stay and to continue for a total of 28 days post discharge as part of the hospital thromboprophylaxis protocol. All patients were followed up for a minimum of 90 days post-surgery; the duration of follow-up was calculated from the date of surgery to the date of the last follow-up, VTE event, or death.

**Figure 1:**
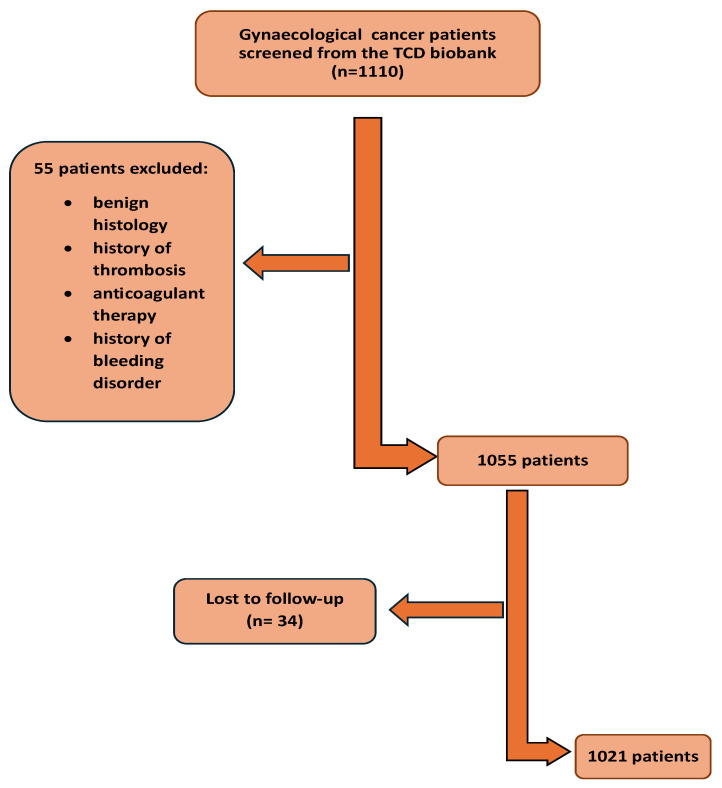
Flow chart of patients on the study.

A total of 1110 patients who met the inclusion/exclusion criteria were identified from the Trinity College Gynaecological cancer bioresource; a total of 55 did not fulfil the inclusion criteria and 34 patients were lost to follow-up (Figure 1). A total of 1021 patients were included in the final analysis.

Patient age, body mass index (BMI), final histological diagnosis, tumour origin, FIGO stage, grade of cancer [25], surgical approach (open/laparoscopic), surgical complexity (calculated according to a modification of the Aletti score (Supplementary Table S1) [26]), duration of hospital stay, chemotherapy (neoadjuvant and adjuvant), and radiotherapy (adjuvant) were recorded. Details of LN status were determined by reviewing surgical notes and histopathology reports; the location and number of LN removed were recorded. The location and number of LN positive for metastasis were recorded from the histology reports.

VTE events were confirmed by documented objective testing (compression ultrasonography, venography, or computed tomography and pulmonary angiogram). Compression sonography is a simple, non-invasive technique that uses ultrasound at a specific frequency to assess for DVT. It involves pressing the ultrasound transducer against the vein to check whether the vein collapses properly and whether there is thrombus in it [27]. Asymptomatic thrombotic events (e.g., PE) detected in computerised tomography to restage cancer pre-chemotherapy or detect the progression of cancer were also included when these events were considered clinically significant.

Demographic and clinical characteristics were summarised using descriptive statistics (Table 1). For each variable, median and inter-quartile range (IQR: 25th to 75th percentiles) and counts (expressed as percentages) were used to describe continuous and categorical variables, respectively. Differences in categorical variables were evaluated in univariate analysis using a chi-squared test, and continuous variables were analysed using an unpaired Student *t*-test.

Univariate analysis (chi-squared) was used to determine the association of LND with postoperative VTE. LN parameters which showed a statistically significant association with VTE from the univariate analysis were taken forward to a multivariate Cox regression analysis to determine hazard ratios (HRs) and the cumulative incidence of VTE adjusted for confounding factors. In all cases, *p* < 0.05 was considered significant. Statistical analysis was performed using SPSS data system version 26 (IBM Corporation, Armonk, NY, USA).

## 3. Results

### 3.1. Patient Demographics

Ovarian cancer was the dominant tumour site with 488 patients, followed by 398 patients with endometrial cancer, 99 patients with cervical cancer, and 36 patients with other types of cancer synchronous pathology. The majority of patients (*n* = 622) underwent open laparotomy and were treatment naïve at the time of surgery. A total of 775 patients received extended thromboprophylaxis. Full demographic details are provided in Table 1.

A total of 41 patients developed VTE in the first 90 days post-surgery. PE was the commonest type of VTE reported in 22 patients, followed by DVT in 14 patients. DVT and PE combined were reported in two patients. The remaining patients had different types of thrombosis, including renal, ovarian vein, right arm, and internal jugular vein thrombosis.

There was a significant association between tumour site and VTE within 90 days post-surgery (*p* = 0.031). Patients with ovarian cancer had the highest rate of VTE with 27 events during the follow-up period (65.8% of VTE events recorded), followed by endometrial cancer patients (21.9%), cervical cancer patients (4.8%), and three patients who had other types of gynaecological cancer including synchronous cancer (7.3%).

Surgical approach, surgical complexity, and duration of hospital stay were all significantly associated with VTE (*p* = 0.0089 < 0.001, <0.001, respectively), with the highest rates in patients undergoing open surgery of intermediate to high complexity with associated longer hospital stay.

Of the 41 patients who developed VTE, 12 patients had surgery prior to 2012 and did not receive extended prophylaxis. A total of 28 patients who developed postoperative VTE were prescribed extended thromboprophylaxis. Extended prophylaxis did not significantly affect VTE rates (*p* = 0.341).

The stage and grade of cancer were also associated with VTE (*p* = 0.022; 0.021); however, BMI and histology were not significantly associated with VTE.

### 3.2. Effect of LND on VTE Rates Post-Surgery

Data on LN status was available in 1006 out of the 1021 patients. A total of 729 patients had pelvic LND, of which 28 (3.84%) developed VTE within 90 days of surgery compared with 13 (4.7%) patients who did not have LND and developed VTE post-surgery.

A total of 176 patients had 1–5 LND. A total of 197 patients had 6–10 dissected LN, and 356 patients had >10 LN removed. There was no significant association between the number of pelvic LN removed and postoperative VTE (*p* = 0.652) (Table 2).

A total of 452 patients underwent para-aortic LND, of which over half had 1–5 nodes removed. Removal of the para-aortic LN was significantly associated with VTE (*p* < 0.001), with the highest rates (14.6%) occurring in patients who had >10 para-aortic LN removed (Table 3).

### 3.3. Lymph Node Metastasis and VTE Post-Surgery

Of the 729 patients who had pelvic LND, 131 patients had pelvic LND positive for metastasis (Table 4). Significantly higher rates of VTE were observed in patients who had more than five pelvic LN positive for metastasis (21.1%) compared with those who had pelvic LND negative for metastasis (3%) (*p* = 0.0000) (Table 4).

**Figure 2:**
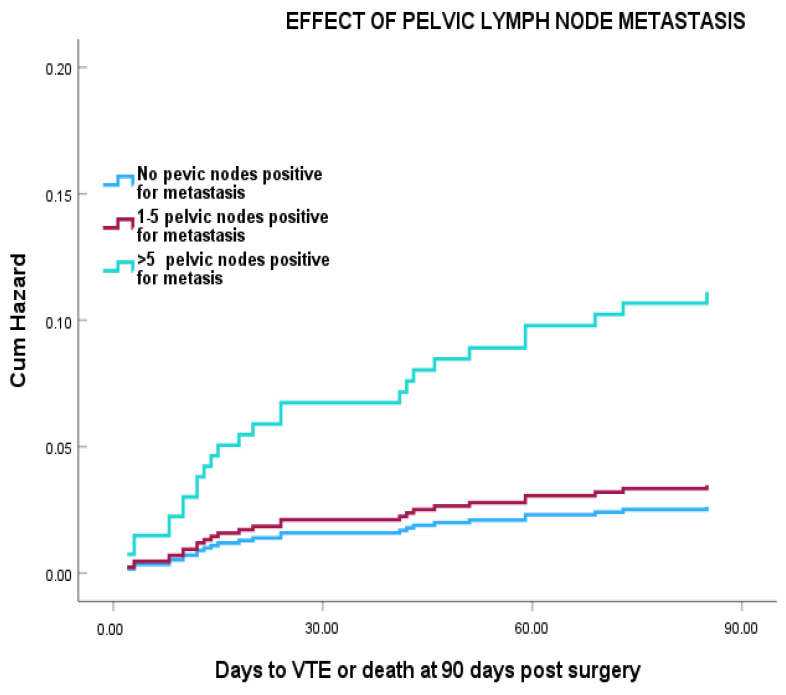
Effect of pelvic lymph node metastasis on VTE risk in gynaecological cancer patients who had pelvic LND (*n* = 701). HR for VTE for HR for >5 lymph nodes positive for metastasis = 7.80 (95% CI 2.64–23.06) (*p* = 0.001). HR = 4.83 (95% CI: 0.99–13.9) following adjustment for age, tumour site, duration of hospital stay, and surgical approach.

Cox regression analysis showed that, patients with more than five positive pelvic LN had a >7-fold increased risk of VTE compared with node negative patients, with the greatest changes occurring in the first 30 days post-surgery (Figure 2), (HR = 7.80 (95% CI 2.64–23.06) (*p* = 0.001). However, this was reduced to HR = 4.83 (95% CI: 0.99–13.9) after adjustment for age, duration of hospital stay, and surgical approach.

A total of 97 (22.2%) of the 452 patients who had para-aortic LND were positive for metastasis (Table 3). Higher rates of VTE (27.3%) were found in those with >5 para-aortic LN positive for metastasis compared with those who had <5 positive for metastasis (*p* = 0.0000).

**Figure 3:**
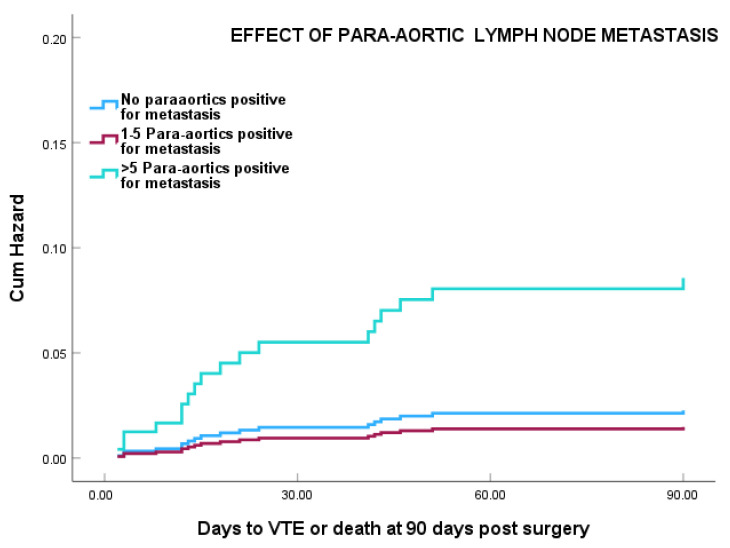
Effect of para-aortic lymph node metastasis on VTE risk in patients who had para-aortic LND (*n* = 434). HR for >5 lymph nodes positive for metastasis = 9.47 (95% CI 2.67–33.6); HR = 3.79 (95% CI 1.44–14.23) following adjustment for age, tumour site, duration of hospital stay, and surgical approach.

Cox regression analysis showed that patients with more than five para-aortic LN positive for metastasis showed a marked increased risk of VTE, particularly in the first 21 days post-surgery at HR 9.47 (95% CI 2.67–33.6) (*p =* 0.0001) (Figure 3). This was still significant at HR = 3.79 (95% CI 1.44–14.23) after adjustment for age, duration of hospital stay, and surgical approach (*p* = 0.011).

## 4. Discussion

LND is a major part of gynaecology cancer surgery, for staging in early endometrial or cervical cancer or debulking in ovarian cancer. This adds to the surgical complexity and increases the risk of VTE, and further treatment planning. Our study found that the site and the number of LND significantly influenced VTE risk in gynaecological cancer patients. Removing >5 para-aortic nodes is associated with the highest risk. In contrast, the removal of pelvic LN was not significantly associated with VTE risk. Additionally, we found that the presence of metastasis in both para-aortic and pelvic lymph nodes was a significant risk factor for VTE even after adjustment for age, duration of hospital stay, and surgical approach.

Our study demonstrates that para-aortic LND is a significant risk factor for VTE. This association can be explained through Virchow’s triad, which encompasses hypercoagulability, venous stasis, and vascular damage [28]. Enlarged para-aortic LN exerts external pressure on vessels, leading to vascular stasis. Additionally, para-aortic LND involves operating near major vessels (aorta and inferior vena cava), which increases the risks of vascular injury, significant bleeding, and prolonged operative time [29]. In a study of patients with endometrial cancer undergoing para-aortic LND, the rate of VTE was notably higher among patients who experienced vascular injury, excessive bleeding, or operative times exceeding 80 min [29].

Our findings confirm the study of Konno et al. who reported 4.9% risk of VTE in patients undergoing pelvic and para-aortic LND compared to 2.2% in patients undergoing only pelvic LND. This may be due to longer operation time (on average 226 min longer) and higher blood loss (~500 mL) in cases including para-aortic LND [30].

Our study further demonstrates that the presence of both pelvic and para-aortic LN metastasis significantly increases the risk of VTE. This elevated risk remains significant even after adjusting for tumour site, patient age, surgical approach, and duration of hospital stay. Metastasis in LN typically indicates an advanced cancer stage, such as stage III or higher, which is directly associated with an increased risk of thrombosis [31]. Advanced cancer stage induces hypercoagulability and causes endothelial damage [32]. Furthermore, LN metastases cause node enlargement, resulting in physical pressure on adjacent vessels and consequent venous stasis [33]. We found an association between the number of metastatic LN and the risk of VTE. Though the effect was noted in both pelvic and para-aortic LND, it was more significant in para-aortic LND.

VTE remains a critical consideration in gynaecological cancer care, particularly in the postoperative setting. The evolution in surgical techniques, such as minimal access surgery and sentinel lymph node sampling, has reshaped clinical practices and raised questions about the necessity of extended thromboprophylaxis in certain patient groups. To address this issue effectively, a better understanding of the pathophysiology of VTE and its associated risk factors in gynaecological cancer is essential for tailoring prophylaxis to individual needs.

These findings provide valuable insights for cancer surgeons, enabling them to identify patients at the highest risk of postoperative VTE and implement targeted prophylactic measures.

There is ongoing controversy regarding the use of extended prophylaxis in minimally invasive surgery (MIS) cases, with no definitive guidelines to support or refute its use, leaving the decision to the surgeon’s judgement [34]. The risk of DVT is thought to be higher than PE in MIS patients who undergo LND [35]. This can be attributed to the steep Trendelenburg position and the extended operative time required for LND, which increases the risk of blood stasis [36]. Lateif et al. reported an increased risk of VTE associated with LND, regardless of the surgical approach, with LND carrying a 1.7-fold increased risk of VTE in gynaecologic cancer patients [20]. However, in prostate cancer, MIS is associated with only one-third the risk of VTE compared with open surgery [21].

In our cohort, the majority of patients who developed VTE were already on extended thromboprophylaxis, suggesting that patients undergoing LND may require either higher doses or longer durations of prophylaxis as suggested by our previous studies [37]. We cannot exclude the possibility that not all patients were compliant with extended prophylaxis, and it was also noted that patients with a lower quality of life were the least likely to adhere to extended prophylaxis. This underscores the importance of careful patient selection and individualised management.

Direct oral anticoagulants (DOACs) can be considered as an alternative to LMWH in patients facing adherence challenges. DOACs are effective for treating VTE in cancer patients and have recently been proposed as a safe alternative to LMWH for extended prophylaxis in high-risk cancer patients, and studies suggest that an oral form of prophylaxis would be preferred by the patient [38]. However, caution is required for elderly patients due to the increased risk of bleeding, particularly if they are also taking non-steroidal anti-inflammatory drugs (NSAIDs), antiplatelets, serotonin–norepinephrine reuptake inhibitors (SNRIs), or selective serotonin reuptake inhibitors (SSRIs) [39].

The interpretation of our study results must account for certain limitations. This was a single-centre retrospective study, and as with all retrospective studies, there are inherent challenges such as missing data. However, it remains the largest published study specifically examining the role of LND and VTE. During the study, from 2006 to 2019, the surgical approach and extent of surgery has evolved, and we cannot exclude that the effects of changes in surgical practice over time may have influenced our results, that the minimal invasive approach is becoming the main surgical approach used, especially in endometrial cancer. Guidelines are constantly being updated, and LND techniques have changed from complete dissection to favouring SLND. Additionally, the possibility that some patients may have experienced asymptomatic VTE, which went undetected, cannot be excluded. We do not routinely screen all patients postoperatively. We could not confirm patient adherence to extended thromboprophylaxis post-hospital discharge. Furthermore, comorbidities were not included in our statistical analysis, and we cannot rule out the possibility that differences in comorbid conditions between the cohorts may have influenced the findings. Future studies should address these limitations to provide a more comprehensive understanding.

## 5. Conclusions

Para-aortic LND is significantly associated with an increased risk of VTE. The presence of metastatic LN further elevates this risk; although determining LN metastasis status intraoperatively remains challenging, we recommend that patients undergoing para-aortic LND receive extended thromboprophylaxis. Further studies are warranted to determine the optimal dose and duration of thromboprophylaxis, as high-risk cohorts may benefit from weight-adjusted dosing to effectively reduce VTE risk.

## Figures and Tables

**Table 1:** Demographics of the population.

		No VTE Within 90 Days (*n* = 980)	VTE Within 90 Days (*n* = 41)	*p* Value
**Age (years) median (IQR)**		590 (49–67)	66 (54–73)	0.045
**Tumour site** Ovary		461 (47.0%)	27 (65.8%)	0.031
Endometrium		389 (39.6%)	9 (21.9%)	
Cervix		97 (9.8%)	2 (4.8%)	
Other		33 (3.3%)	3 (7.3%)	
**Histology** Clear cell		36 (3.6%)	1(2.4%)	0.082
Serous		289 (29.4%)	23 (56.1%)	
Mucinous		32 (3.2%)	0 (0%)	
Endometrioid (ovarian)		41 (4.1%)	1 (2.4%)	
Endometrial adenocarcnoma		300 (30.6%)	6 (14.6%)	
Squamous		69 (7.0%)	2 (4.87%)	
Sarcoma		12 (1.2%)	0 (0%)	
Mixed		45 (4.6%)	3 (7.3%)	
Borderline		63 (6.4%)	1 (2.4%)	
Other		93 (9.4%)	4 (9.7%)	
**Stage** I		511 (52.1%)	11 (26.8%)	0.022
II		74 (7.5%)	2 (4.8%)	
III		244 (24.8%)	18 (43.9%)	
IV		112 (11.4%)	8 (19.5%)	
Recurrent		36 (3.6%)	2 (4.8%)	
N/A		1 (0.1%)	0 (0%)	
Missing		2 (0.2%)	0 (0%)	
**Grade** I		288 (29.3%)	3 (7.3%)	0.020
II		196 (20.0%)	7 (17.1%)	
III		392 (40.0%)	26 (63.4%)	
N/A		104 (10.6%)	5 (12.1%)	
**BMI** >30 kg/m^2^		362 (37%)	16 (39.0%)	0.97
<30 kg/m^2^		548 (55.9%)	24 (58.5%)	
Missing		70 (7.1%)	1 (2.4%)	
**Extended prophylaxis**	Yes	747 (76.2%)	28 (68.3%)	0.341
	No	229 (23.3%)	12 (29.2%)	
	N/A	4 (0.4%)	1 (2.4)	
**Chemotherapy** Neoadjuvant		125 (12.7%)	8 (19.5%)	0.025
Adjuvant		332 (33.8%)	20 (48.5%)	
No chemotherapy		522 (53.2%)	13 (31.7%)	
Missing		1 (0.1%)	0 (0%)	
**Radiotherapy** Yes		283 (28.8%)	5 (12.1%)	0.108
No		694 (70.6%)	36 (87.8%)	
Missing		3 (0.3%)	0 (0.0%)	
**Surgical Complexity** Low		381 (38.8%)	11 (26.8%)	<0.001
Intermediate		506 (51.6%)	15 (36.5%)	
High		90 (9.1%)	15 (36.5%)	
Missing data		3 (0.3%)	0 (0.0%)	
**Surgical Approach** Open		589 (60.1%)	33 (80.4%) 8 (19.5%)	0.009
Laparoscopic		390 (39.7%)		
Missing		1 (0.11%)		
**Duration of hospital stay (median days (IQR))**		9.8 (2–120)	20.6 (5–70)	<0.001
**History of other cancers**		130 (13.2%)	9 (23.3%)	0.115

**Table 2:** Relationship between the number of pelvic lymph nodes removed and risk of VTE within 90 days of surgery.

	No VTE Within 90 Days Post-Surgery (*n*)	VTE Within 90 Days Post-Surgery (*n*)	Total (*n*)	*p* Value
**No pelvic nodes removed**	264 (95.3%)	13(4.7%)	277	0.652
**1–5 Pelvic nodes removed**	169 (96.0%)	7 (4.0%)	176	
**6–10 Pelvic nodes removed**	192 (97.5%)	5 (2.5%)	197	
**>10 pelvic lymph node removed**	340 (95.5%)	16 (4.5%)	356	

**Table 3:** Relationship between the number of para-aortic lymph nodes removed and risk of VTE within 90 days of surgery.

	No VTE Within 90 Days Post-Surgery (*n*)	VTE Within 90 Days Post-Surgery (*n*)	Total	*p* Value
**No para-aortic nodes removed**	531 (95.8%)	23 (4.2%)	554	0.001
**1–5 para-aortic nodes removed**	296 (97.0%)	9 (3.0%)	305	
**6–10 para-aortic nodes removed**	103 (97.2%)	3 (2.8%)	106	
**>10 para-aortic nodes removed**	35 (85.4%)	6 (14.6%)	41	

**Table 4:** Relationship between the number of pelvic lymph node metastasis and risk of VTE within 90 days of surgery.

		No VTE Within 90 Days Post-Surgery (n)	VTE Within 90 Days Post-Surgery (n)	Total (n)	*p* Value
**Total number of patients with pelvic nodes removed (n)**		701	28	729	
**Negative for metastasis (n)**		580 (97.0)	18 (3.0)	598	0.000
**1–5 pelvic nodes positive for metastasis**		106 (94.6)	6 (5.4)	112	
**>5 pelvic nodes positive for metastasis**		15 (78.9)	4 (21.1)	19	

## Data Availability

The raw data supporting the conclusions of this article will be made available by the authors on reasonable request.

## Supplementary Materials

The following supporting information can be downloaded at: https://www.mdpi.com/article/10.3390/cancers18010040/s1, Table S1: Surgical classification and complexity score group modified from Aletti et al; Patient information leaflet; Patient consent.
