# Supplementary material for: Para-Aortic Lymph Node Dissection and Metastasis Increase the Rate of Postoperative VTE in Gynaecological Cancers
Source: Cancers (Basel). 2025 Dec 22;18(1):40. doi: 10.3390/cancers18010040 (PMC12784709; doi:10.3390/cancers18010040)
Supplement: Supplementary file 1 [file cancers-18-00040-s001.zip › cancers-4018740-supplementary.pdf]

Supplementary

Supplementary I: Patient information leaflet

Title: A study to identify protein/micro-RNA patterns and blood coagulation proteins in gynaecological cancers.

MicroRNA, protein and Tissue factor (TF) research in your blood

You are invited to participate in an optional study called ‘the microRNA, protein and blood coagulation study’ that involves looking at small pieces of genetic material called microRNAs and proteins including those involved in blood clotting in samples of your blood and tissue.

What is the ‘the microRNA, protein and TF study’ and why is it being done?

We all have microRNAs in our body. MicroRNAs are small pieces of genetic material, which affect how the bigger genes in cells work. They can change the way cells react to drugs and might explain why certain diseases occur in people. Gynaecological cancers are difficult to diagnose, studying proteins in the bloodstream might help us develop tests which may identify women who have cancer at an early stage. TF is a protein that is involved in blood clotting. By measuring proteins which help in blood clotting we hope to find out why some patients have an increased the risk of blood clots after surgery. To do this we need to obtain samples from normal healthy women undergoing

hysterectomy as well as women undergoing surgery for possible gynaecological cancer.

Joining the study is optional. This means that you may

• Choose to join but then change your mind before your sample is taken or at any time during the study

• Chose not to join

What will happen if I decide to participate?

If you decide to join the study we will draw a blood sample before your surgery, and a small piece of tissue will also be taken during your surgery. This will be about an extra 2 dessert spoonfuls of blood, the blood and tissue samples taken as part of this study will be taken to the academic research laboratories in St. James's Hospital. In some cases we will also draw additional blood samples 5 days after surgery (when you are still in hospital) and when you return to the hospital between (2 - 6 weeks after surgery) for your check-up. The samples will be coded and will not be labelled with any information that directly identifies you. The connection between the code and you will only be kept by the study doctor. Your samples may be kept between 2 and 15 years for these tests. Any samples remaining will be destroyed after this time. The only tests that will be done are those described in this form and no other tests will be done without additional consent from you. The tests on your blood will not give any information about the diseases that are passed on in families or any diseases that you may develop in the future. Your study doctor will answer any questions which you may have. Your blood samples will be given the same code as your other study information and kept in locked storage. Anyone who

works with your samples will hold this information and results in confidence. The academic research group that your doctor is a member of may store your blood samples for up to 15 years after the end of the study .

What will happen if I decide not to participate?

You will receive all your usual care, including blood tests necessary for your surgery and follow-up care. We will not take any blood samples to use in this or any other study without your permission.

What if I change my mind?

If you choose to stop participating in the study after giving a sample we will destroy it within 30 days. If your sample is being processed we will have to wait until all steps are done before we can destroy it. This might be longer than 30 days. The academic research group will not use your data for analysis.

What side effects or risk can I expect from giving a blood sample?

When you give blood, you may feel faint, or experience mild pain, bruising, irritation or redness at the site. In rare cases, you may get an infection.

What benefits can I expect from the microRNA, protein and blood coagulation study?

If you take part in the study and agree to give samples, you will not benefit directly. However, the results may help scientists and doctors understand how gynaecological cancers arise and how to detect them at an earlier stage, it will also help doctors to develop new tests to identify patients at risk of blood clots. This may mean better treatment for patients in the future.

Supplementary II: Patient consent:

Please sign both copies

CONSENT FORM

CONSENT FORM FOR PARTICIPATION IN GENETIC RESEARCH

Protocol Number: ……………………………………..

Participant Identification Number: ……………………………………

Title of Protocol: ……………………………………

Name of Institution leading the Research : Trinity College Dublin………………

Research Director: Prof John O'Leary, Dr Sharon O'Toole,

Dr Noreen Gleeson, Dr Tom D'Arcy

Phone Number and Contact Details: 01-8962106 Sharon O'Toole..………

Please initial boxes

1. I have read the attached information sheet on the above project dated………… ☐

and have been given a copy to keep. The information has been fully explained to me

and I have had an opportunity to ask questions about the project and understand

why the research is being done and any foreseeable risks or consequences involved.

I also understand that no guarantee can be given about the possible results.

2. I agree to give a sample(s) of ☐

blood / other bodily sample / DNA for research in the above project.

I understand how the sample will be collected, that giving a sample for this research

is voluntary and that I am free to withdraw my approval for use of the sample at any

time without giving a reason. If I withdraw my consent I understand that my sample

will be destroyed unless I otherwise authorise. I understand that I may ask for my

samples to be destroyed and that this will be without my medical treatment or legal

rights being affected. I agree that the samples I have given and the information

gathered by me can be stored and looked after by the (Trinity College Dublin).

I understand that any genetic information obtained will / will not be made available

to me.

3. I give permission for my medical records to be looked at and information ☐

taken from them to be analysed in the strictest confidence by the relevant and

responsible people from the (DISCOVARY consortium) or from organisations

supervising the research. I have been told that all medical information / data

pertaining to me will be protected by the principles of confidentiality

and both national and E U data protection legislation. I have further been told of /

shown assurances that this also applies to all medical information / data pertaining to

me that are utilised in any non-E U state.

4. I understand that the confidentiality of the sample(s) I donate and ☐

information derived therefrom will be protected. I have been told that all medical

information / data pertaining to me and derived from the sample(s) will be protected

by the principles of confidentiality and both national and E U data protection

legislation. I have further been told of / shown assurances that this also applies to all

medical information / data pertaining to me and derived from the sample(s) that are

utilised in any non-E U state.

FOR OTHER GENETIC RESEARCH :

(Note : New research should be submitted for approval by the Research

Ethics Committee before proceeding)

5. I understand that future research using the sample I give may include genetic

research aimed at understanding the genetic influences in disease but that such test

may not be of predictive / clinical value and that the results of these investigations

are unlikely to have any implications for me personally. In the event a test is of

predictive or clinical value, my healthcare team will be informed.

☐

6. I understand that I will not benefit financially in any way if this research leads to

the development of a new treatment or medical test.

☐

7. I know how to contact the research team if I need to.

☐

………………………………….. ……….. …………………   ……………………
…………………………

Name of participant (BLOCK CAPITALS) Date Signature

……………………………….. ……. ……….   ………………………………
…………………………..

Name of researcher Date Signature

……………………………….. …………..   ………………………………… ….………………………

Name of witness   Date   Signature

Supplementary III:

Supplementary table S1: Surgical classification and complexity score group modified from Aletti et al.

Points	Procedure
1	Hysterectomy +/-salpingoophorectomy
3	Radical Hysterectomy
1	Omentectomy
1	Pelvic lymphadenectomy
2	Para aortic lymphadenectomy
1	Pelvic &/Or abdominal peritoneal stripping
3	Recto sigmoid resection with anastomosis
2	Large bowel resection
1	Small bowel resection
2	Diaphragmatic stripping
2	Splenectomy
2	Liver resection
2	Radical Vulvectomy
2	Groin dissection
1	Simple vulvectomy
1	Vaginal Hysterectomy
3	Trachelectomy
**Points**	**Complexity score group**

**Points**	**Procedure**
3or less	Low
4 to 7	Intermediate
8 or more	High
